# Influence of Employments upon Health

**Published:** 1844-02-10

**Authors:** 


INFLUENCE OF EMPLOYMENTS UPON HEALTH.

The following summary embodies the conclusions to be drawn from an elaborate and valuable paper, published by Dr. Guy, in the last number of the Journal of the Statistical Society.

1. In females, the ratio of cases of pulmonary consumption to those of all other diseases, is highest in those following sedentary employments, less in those having mixed in-door occupations, and least of all in those employed out of doors. The highest ratio occurs in the case of females whose habits of life are irregular (prostitutes).

2. In men the ratio of cases of pulmonary consumption to those of all other diseases, is somewhat higher in those following in-door occupation than in those working in the open air

3. The ratio of cases of pulmonary consumption to those of all other diseases, in the case of men following in-door employments, varies inversely as the amount of exertion, being highest where there is least exertion, and lowest in employments requiring strong exercise.

4. Neither a constrained posture, nor exposure to a high temperature, nor a moist atmosphere, appears to have any marked effect in promoting pulmonary consumption.

5. The ratio of cases of pulmonary consumption to those of all other diseases, is highest in the case of men whose employments expose them to the inhalation of dust, there being in persons so employed two cases of consumption for less than three of all other diseases.

6. The ratio is also high in the case of persons exposed to habits of intemperance, there being two cases of pulmonary consumption to five of all other diseases.

7. The age at which pulmonary consumption makes its attack, varies with the employment, being earlier in those occupations characterised by a high ratio of consumptive cases. Thus it is earlier in those following in-door occupations than in those employed in the open air, and in those using little exertion than those using much. It also occurs very early in those exposed to the temptation of intemperance, and in those whose occupations lead to the inhalation of dust.—*Prov. Med. Jour.* Nov, 1843.

